# A simulated Northern Hemisphere terrestrial climate dataset for the past 60,000 years

**DOI:** 10.1038/s41597-019-0277-1

**Published:** 2019-11-07

**Authors:** Edward Armstrong, Peter O. Hopcroft, Paul J. Valdes

**Affiliations:** 1 School of Geographical Sciences, University of Bristol, Bristol, UK; 2 Cabot Institute, University of Bristol, Bristol, UK; 3 School of Geography, Earth and Environmental Sciences, University of Birmingham, Edgbaston, B15 2TT UK

**Keywords:** Palaeoclimate, Projection and prediction, Climate and Earth system modelling

## Abstract

We present a continuous land-based climate reconstruction dataset extending back 60 kyr from 0 BP (1950) at 0.5° resolution on a monthly timestep for 0°N to 90°N. It has been generated from 42 discrete snapshot simulations using the HadCM3B-M2.1 coupled general circulation model. We incorporate Dansgaard-Oeschger (DO) and Heinrich events to represent millennial scale variability, based on a temperature reconstruction from Greenland ice-cores, with a spatial fingerprint based on a freshwater hosing simulation with HadCM3B-M2.1. Interannual variability is also added and derived from the initial snapshot simulations. Model output has been downscaled to 0.5° resolution (using simple bilinear interpolation) and bias corrected. Here we present surface air temperature, precipitation, incoming shortwave energy, minimum monthly temperature, snow depth, wind chill and number of rainy days per month. This is one of the first open access climate datasets of this kind and can be used to study the impact of millennial to orbital-scale climate change on terrestrial greenhouse gas cycling, northern extra-tropical vegetation, and megaflora and megafauna population dynamics.

## Abstract (summary)

**Table:** 

Measurement(s)	climate change • temperature of air • precipitation process • radiation • snow • water-based rainfall
Technology Type(s)	computational modeling technique • digital curation
Factor Type(s)	Year
Sample Characteristic - Environment	climate system
Sample Characteristic - Location	Northern hemisphere

Machine-accessible metadata file describing the reported data: 10.6084/m9.figshare.10012859

## Background & Summary

The last glacial period witnessed significant fluctuations in global climate, in the long term driven predominantly by orbital changes but with additional more rapid millennial scale fluctuations. This period encapsulated the last glacial maximum (LGM), when temperatures were potentially between 19–22 °C cooler across Greenland^1^, resulting in expansion of ice cover and consequent polar amplification^2^. This lowered sea level by up to 127–135 m^3^, impacted ocean circulation^4^ and opened previously inundated land bridges such as the Bering Straits^5^, potentially influencing the migration of animal species including humans^6^. Prior to the LGM, the climate was cold but was punctuated with a high degree of millennial variability. Since the LGM the climate has warmed, sea-level fallen and the climate in more recent times been influenced by anthropogenic activity. Reconstructing climate change over this period has important uses in a wide range of academic research.

Palaeoclimate observational datasets obtained in the field from ice or sediment-cores and consisting of timeseries of variables such as isotopes of carbon, oxygen, hydrogen or nitrogen, sedimentary input etc., are invaluable for understanding the spectrum of climatic variations^7–10^. These records have provided evidence for glaciations, abrupt climate change and more. Although they constitute direct measurements, many proxies may respond to multiple climatic variables or even non-linear combinations of variables. Moreover, for many reconstructed climate variables, there remains limited spatial coverage, especially as we move beyond time-slices such as the mid-Holocene or LGM, to transient experiments. Climate models offer an alternative method to study past climates. Although these are subject to their own wide range of biases, the benefit of such an approach is the ability to produce a high frequency dataset with global coverage for a full range of climate variables, all tied together in a manner that is consistent with the underlying physics encapsulated by the model.

Using climate models in palaeoclimate studies often focuses on key climate periods, such as the LGM^11–13^. Utilising a model for simulating millennial scale time periods is computationally expensive, so past studies that have generated a continuous climate timeseries have utilised lower resolution models, such as Earth System Models of Intermediate Complexity (EMICs) or Energy Balance models (EBMs)^14,15^. Studies that have utilised general circulation models (GCMs) have used low-resolution versions^16^, utilised a simple slab-ocean^17^, or accelerated the boundary conditions^18^. Producing a global timeseries using a fully coupled complex climate model is currently very challenging due to the extremely long run times involved, as well as the difficulty in storing such extensive model output. In order to avoid these issues, a ‘snapshot’ approach can be used, where model output is generated at intervals over the period under analysis. Each simulation uses pre-defined boundary conditions, including greenhouse gases, orbital parameters, extent of ice sheet cover and sea level. The key assumption is that the climate is in equilibrium with the boundary conditions, and that the final climate is largely independent of the initial conditions (so that the simulations can be run in parallel). Though both of these assumptions can be challenged, experience has shown that the climate outputs are a good representation of the orbital time scale climate change^2,19^.

For some impact analyses, such as simulating peat formation^20^, niche modelling^21^, and species distribution modelling^22^, these snapshot simulations may be sufficient. However, the millennial variability of climate may also be of importance^23^. Hence we need to link these climate snapshot outputs together and incorporate cycles of higher-frequency variability, as they are necessarily omitted by the sampling frequency of the initial GCM ‘snapshots’. Observed millennial scale climate variability includes climate perturbations such as the Dansgaard-Oeschger (D-O) and Heinrich (HE) events^24–27^. The last glacial cycle is characterised by 25 abrupt DO events, with consequent warming of between 8 °C to 16 °C over Greenland^10,25^. Although the mechanisms responsible for these events are not fully understood, they are thought to be driven by abrupt changes in Atlantic meridional overturning circulation (AMOC) strength, possibly due to perturbations in the freshwater budget due to the melting of icebergs and sea-ice fluctuations^28,29^. Imprinted on these millennial scale fluctuations is higher frequency inter/intra-annual and seasonal variability, including internal climate oscillations, driven by the different response times and non-linear interactions within the climate system. Recent work has suggested that inter-annual variability changes depending on the climate regime^30^.

Here we present a monthly climate timeseries for the Northern Hemisphere (0°N–90°N) land surface, generated from 42 snapshot simulations of the past 60 kyrs at either 1 kyr or 2 kyr intervals using the Bristol University version of the fully coupled global circulation model HadCM3, termed HadCM3B-M2.1^31^. We present the results for terrestrial grid-points only, as this dataset is aimed primarily towards terrestrial ecosystem modellers such as those investigating vegetation and species population dynamics, although the dataset can be used for a wide range of research. We focus specifically on the Northern Hemisphere, as we have not incorporated a model for how the two hemispheres behave during abrupt events (i.e. the bipolar see-saw). Millennial scale variability is added by incorporating the spatial results of hosing experiments, which simulate a change in the strength of the AMOC analogous to a D-O event. This is then combined with a temperature reconstruction from Greenland ice-core derived from nitrogen isotopes. Inter-annual variability has been incorporated directly from the model output. Finally the data has been downscaled to 0.5° resolution and bias corrected using the Climate Research Unit (CRU) data^32^.

## Methods

### The HadCM3B-M2.1 coupled climate model

The Hadley Centre Coupled Model 3 Bristol (HadCM3B) is a coupled climate model consisting of a 3D dynamical atmosphere^33^ and ocean^34^ component. HadCM3B is a version of the more commonly known HadCM3 that has been developed at the University of Bristol, and is outlined in detail in the study of Valdes *et al*.^31^. It differs slightly to the original model code of HadCM3^33,34^ as it has undergone a number of minor bug fixes as documented in detail in Section 2.1 of Valdes *et al*.^31^, although such changes have been shown to have only a minimal impact on the simulated climate.

Despite the relatively old age of HadCM3B, the model has been shown to produce an accurate representation of different climate variables and remains competitive with other more modern climate models used in CMIP5^31^. A key advantage of the model is that it is computationally fast, which permits long (i.e. millennial) scale simulations and large ensemble studies.

The atmosphere component of HadCM3B^33^ has a resolution of 3.75° × 2.75° (equivalent to 96 × 73 grid points) and 19 vertical levels with a timestep of 30 minutes. The ocean model^34^ has a resolution of 1.25° × 1.25° (equivalent to a 288 × 144 grid points) with 20 vertical levels and a timestep of one hour. The levels exhibit a finer resolution towards the surface, the first having a thickness of 10 m and the deepest a thickness of 616 m.

HadCM3 incorporates the land-surface scheme MOSES (Met Office Surface Exchange Scheme) that models the fluxes of energy and water and the physiological processes of photosynthesis, transpiration and respiration which is dependent on stomatal conductance and CO_2_ concentration^35^. Here we use MOSES 2 version 2.1 (v2.1), therefore the full model name is HadCM3B-M2.1. For a full overview of MOSES 2 see Essery *et al*.^36^ and for the differences between MOSES v2.1 and v2.2 see Valdes *et al*.^31^. MOSES 2 incorporates the fractional coverage of nine different surface types, which are simulated by the dynamic global vegetation model (DGVM) TRIFFID. The vegetation can dynamically evolve throughout the simulations depending on four variables; moisture, temperature, atmospheric CO_2_^35^, and competition between plant functional types (PFTs).

Sea ice is simulated via a zero-layer model^37^ and is calculated on top of the ocean grid with movement controlled by the ocean currents in the upper ocean^34^. Ice is formed in leads (i.e. the fractures that form due to stresses) and by snowfall, and removal occurs from the base continually throughout the year and on the surface via melting in the summer. The salinity of sea-ice is assumed to be constant with a flux into the ocean depending on melting or ice formation.

The model does not include an interactive ice model, or carbon and methane cycle so these boundary conditions have been imposed (see next section).

### Experimental set-up

#### Snapshot simulations and boundary conditions

**Fig. 1:**
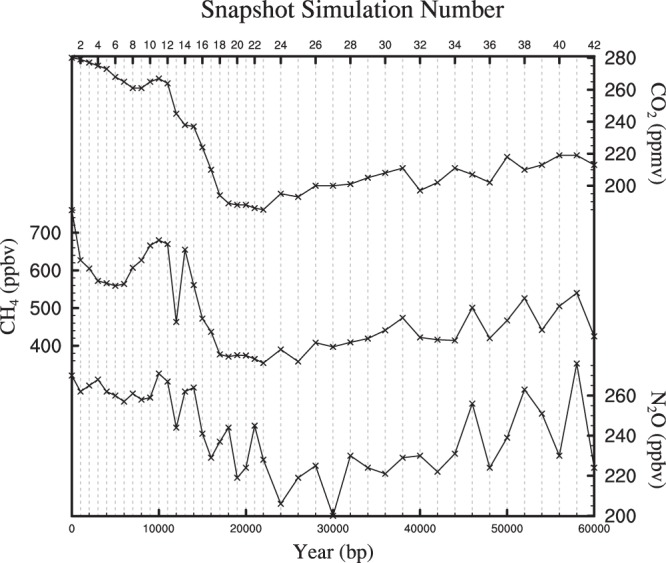
Prescribed greenhouse gases for the 42 snapshot simulations. Each vertical line represents the position of the snapshot simulations in the 60 kyr timeseries.

This study incorporates the results from 42 ‘snapshot’ simulations which are updated versions of those outlined in Singarayer and Valdes^2^ and Davies-Bernard *et al*.^33^. Each simulation has been forced with prescribed variations in orbital parameters which are very well constrained^14^, greenhouse gases (see Fig. 1) and ice-sheets. A summary of these is given in Table 1. Concentrations of atmospheric CO_2_ are from the Vostok Ice core^38,39^ whilst N_2_O and CH_4_ concentrations are taken from the EPICA Dome C ice core^40^. The snapshot simulations extend back to 60 kyr before present (BP), where 0 BP refers to the year 1950. The 0 BP simulation has greenhouses gases that represent the pre-industrial (PI) period, where PI refers to 1850, equivalent to a CO_2_ concentration of 280 ppm. This simulation therefore represents a 1950 world in which greenhouse gases have not risen relative to the pre-industrial.

**Table 1:** Names, years and boundary conditions for the 42 snapshot simulations used in this study.

Simulation	Simulation Name	Year kyr	Orbital Parameters			C02 (ppm)	CH4 (ppbv)	N20 (ppbv)
			Eccentricity	Obliquity (°)	Precession			
1	teiia	0	0.01724	23.446	0.017	280	760	270
2	teiib	1	0.01764	23.573	0.018	279	627	262
3	teiic	2	0.01802	23.697	0.017	277	605	265
4	teiid	3	0.01838	23.815	0.014	275	572	268
5	teiie	4	0.01870	23.923	0.010	273	566	262
6	teiif	5	0.01899	24.019	0.005	268	559	260
7	teiig	6	0.01925	24.100	0.000	265	564	257
8	teiih	7	0.01948	24.163	−0.006	261	607	261
9	teiii	8	0.01967	24.206	−0.011	261	627	258
10	teiij	9	0.01984	24.229	−0.015	265	666	259
11	teiik	10	0.01997	24.229	−0.018	267	680	271
12	teiil	11	0.02007	24.207	−0.020	264	670	267
13	teiim	12	0.02014	24.161	−0.020	245	463	244
14	teiin	13	0.02018	24.093	−0.018	238	655	262
15	teiio	14	0.02018	24.004	−0.015	237	561	264
16	teiip	15	0.02015	23.895	−0.011	224	472	241
17	teiiq	16	0.02010	23.769	−0.005	210	437	229
18	teiir	17	0.02001	23.627	0.000	194	377	237
19	teiis	18	0.01990	23.475	0.006	189	371	244
20	teiit	19	0.01976	23.315	0.011	188	375	219
21	teiiu	20	0.01959	23.151	0.015	188	374	224
22	teiiv	21	0.01940	22.989	0.018	186	365	245
23	teiiw	22	0.01918	22.832	0.019	185	354	228
24	teiix	24	0.01869	22.553	0.017	195	390	206
25	teiiy	26	0.01814	22.348	0.010	193	358	219
26	teiiz	28	0.01753	22.244	0.000	200	408	225
27	teiiA	30	0.01690	22.255	−0.009	200	397	200
28	teiiB	32	0.01627	22.382	−0.015	201	409	230
29	teiiC	34	0.01567	22.610	−0.016	205	419	224
30	teiiD	36	0.01513	22.913	−0.013	208	441	221
31	teiiE	38	0.01467	23.257	−0.007	211	474	229
32	teiiF	40	0.01433	23.605	0.000	197	422	230
33	teiiG	42	0.01413	23.922	0.007	202	416	222
34	teiiH	44	0.01409	24.178	0.011	211	414	231
35	teiiI	46	0.01422	24.353	0.014	207	501	256
36	teiiJ	48	0.01450	24.433	0.014	202	420	224
37	teiiK	50	0.01493	24.416	0.011	218	467	239
38	teiiL	52	0.01550	24.305	0.005	210	526	263
39	teiiM	54	0.01617	24.113	−0.002	213	442	251
40	teiiN	56	0.01693	23.859	−0.010	219	505	230
41	teiiO	58	0.01776	23.565	−0.016	219	540	276
42	teiiP	60	0.01865	23.258	−0.019	213	425	224

Due to the lack of an ice-sheet model, the extent and elevation of ice-sheets has also been imposed. The major regions are the Antarctic, Greenland, North American and Fennoscandian ice sheets, which impact isotactic rebound and sea level. Here, reconstructions from present to the LGM (21 kyr BP) have been based on the ICE-5G model^41^. This gives the evolution of a number of variables including ice extent, thickness and isostatic rebound on 500-year intervals over this period. Within the model these are used to calculate bathymetry, continental elevation (from ice sheet thickness and rebound), ice extent, and the land-sea mask at each time interval.

Beyond the LGM to 60 kyr BP, there have been few studies that have attempted to reconstruct ice-sheets (and less data is preserved due to the LGM ice sheet removing evidence of previous ice) and so data is less well constrained. We have experimented with two methodologies. The first used the ice sheet that the ICE-5G model uses to spin-up. The method assumes that during glacial periods land ice coverage is similar to that at the LGM whereas thickness is defined by the δ^18^O record of Martinsen *et al*.^42^. This method was used in Singarayer *et al*.^2^, but likely overestimates the area of the ice sheet which then overestimates the albedo effect of the ice. An alternative method equates earlier (pre-LGM) ice sheets to the equivalent ice volume (sea level) during the deglaciation. For instance, the sea level depression at 40 kyr BP is compared to the sea level during the deglaciation. Where these are the same, the ice-sheet extent is inferred to be the same as that at 40 kyr BP. This approximation is imprecise because ice sheets show different structures during growth and decay phases, but it is a much better approximation to the ice area than the previous method. For this reason, we use these in the current simulations (as in Davies-Barnard, *et al*.^43^).

The boundary conditions have been incorporated into 42 snapshot simulations, which are set at 1000-year intervals between 0 and 22 kyr BP and 2000-year intervals between 22 to 60 kyr BP. Each simulation has been run for 2000 years of spin-up, and initialised from previous simulations for each period that have been run under the same boundary conditions, albeit with the addition of dynamic vegetation. Each simulation has therefore been run for a minimum of 6000 years. This permitted the experiments to be run simultaneously and hence is highly efficient, taking just a month or two on a high performance computer. A fully time continuous simulation would have taken about 3 years. Analysis has been conducted on the final 1000 year climatologies of each simulation unless stated otherwise.

**Fig. 2:**
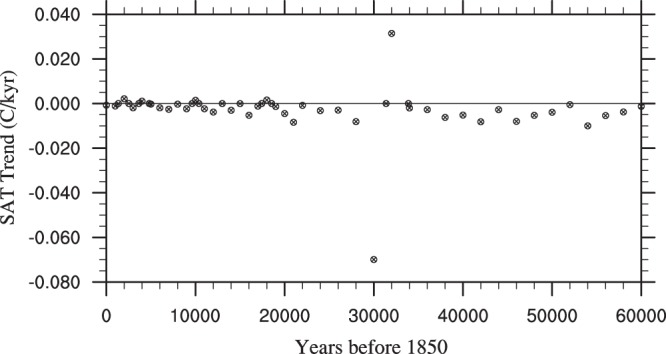
The average trend in SAT (°C/kyr) for the 42 snapshot simulations. These have been calculated from the final 1000 years of each of the 2000 year simulations and highlight their equilibrium state.

In order to show the equilibrium state of the 42 simulations, Fig. 2 shows the linear trend in surface air temperature for the 42 simulations. The linear trends in surface temperature are small, in most cases less than 0.01 °C/millennium. Although a trend remains, models commonly need to be run for many thousands of years to be in complete equilibrium, so we determine this to be suitable.

#### Splining

The snapshot climatologies for each of 42 simulations were splined to a monthly time-series. Here the average climatology for each month for each simulation were splined together, producing a dataset of 60,000 × 12 time points. This approach is applied to both climate variables, including the land sea mask and the ice fraction used in each snapshot GCM simulation. The land sea mask and ice fraction are subsequently rounded to 0 or 100% coverage in any gridcell in every year.

**Fig. 3:**
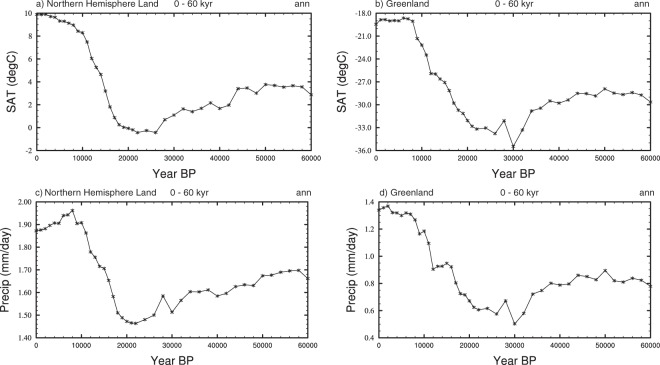
Timeseries showing the splined data. These have been generated from the annual averages from the final 1000 years of the snapshot simulations. (**a**) Northern Hemisphere SATs (°C). (**b**) Greenland SATs (°C), Northern Hemisphere precipitation (mm/day), and (**d**) Greenland precipitation (mm/day).

Splining has been done using the NCAR command language (NCL) ftcurv function. This produces a smooth curve for a variable between each of the simulations using a technique termed spline under tension^44^. The resultant timeseries for mean annual surface air temperature (SAT) and precipitation for the northern extra tropics and Greenland is shown in Fig. 3.

#### Interannual variability

**Fig. 4:**
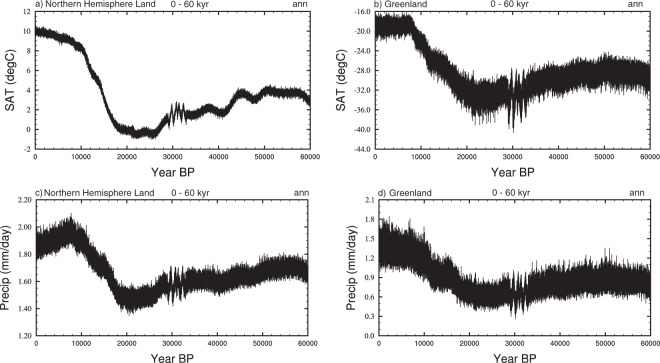
Timeseries showing the addition of interannual variability to the splined data. Interannual variability has been taken from the final 1000 years of the model simulations and switches at the mid point of each of the snapshot simulations. (**a**) Northern Hemisphere SATs (°C). (**b**) Greenland SATs (°C), Northern Hemisphere precipitation (mm/day), and (**d**) Greenland precipitation (mm/day).

Interannual variability is incorporated in order to account for high frequency internal climate variability. A 1000-year timeseries of variability is calculated from the final 1000 years of each of the model snapshot simulations, by subtracting the climatological mean from the timeseries. This is then incorporated into the 60 kyr splined dataset, with variability switching at the mid-point between two simulations. Where the snapshot simulations are at 2000-year intervals (beyond 22 kyr BP), the 1000-year sections are repeated twice up to the mid-point between two of the simulations, at which point the variability switches to repeating the 1000-year segment from the subsequent simulation. The addition of variability to the splined data for SATs and precipitation is shown in Fig. 4.

**Fig. 5:**
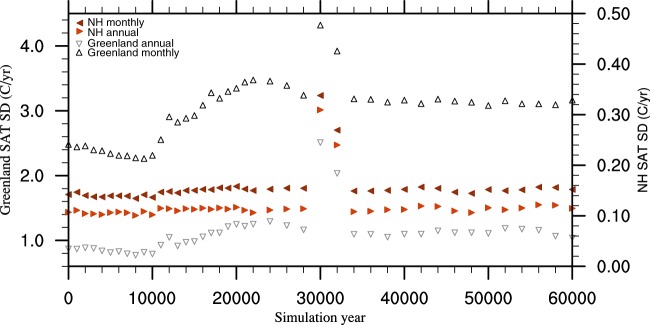
Standard deviations (SD) for the interannual variability component applied to the splined data for each of the 42 snapshot simulations. Annual and monthly values are given for the Northern Hemisphere and Greenland.

Although this is an idealised approach, it does give an indication as to the extent of high frequency stochastic internal climate variability simulated by a climate model, and how this might vary over time. Note that variability on this timescale is an artefact of the model and is therefore synthetic, rather than being a product of data assimilation. Figure 5 shows the monthly and annual standard deviations for high-frequency variability for the Northern Hemisphere and Greenland. Northern Hemisphere variability remains relatively constant throughout the simulations, with the exception of 30 and 32 kyr BP. In contrast, Greenland shows an increase in variability after the Holocene. Past studies utilising proxy records have also highlighted an increase in variability between the Holocene and the LGM^30,45^, although we do not see this here on a hemispheric scale. This may be linked to an increase in the meridional temperature gradient at the LGM which increases the extent of variability^30^.

There is a clear change in variability between 29 kyr and 33 kyr BP. This represents four 1 kyr repeating sections, two of which from the 30 kyr and 32 kyr BP simulations respectively. These intra-millennial oscillations are an interesting artefact of these two model simulations, the direct cause of which is uncertain. They may reflect a long-term, millennial scale oscillation present in the simulations, a phenomenon which may represent a salt oscillator as previously identified by Peltier and Vettoretti (2014) in the CESM model^46^. However, further analysis is required to test what drives this in HadCM3B. The increase in variability between 30–32 kyr BP is largely masked following he addition of DO-variability as discussed in the following section.

#### Millennial scale climate variability

The next step is to incorporate abrupt, millennial scale variability, which specifically refers to the typical D-O cycles that populate the glacial period. These tend to begin with a rapid (i.e. decadal) warming event which then cools over a longer period of time before cooling more rapidly towards the baseline. A number of studies have hypothesised that these events are driven by fluctuations in the strength of the AMOC, as indicated by ice-cores and sediment proxies^24–27^. Commonly therefore, modelling such events utilises hosing experiments which impact the strength of the AMOC^47^.

**Fig. 6:**
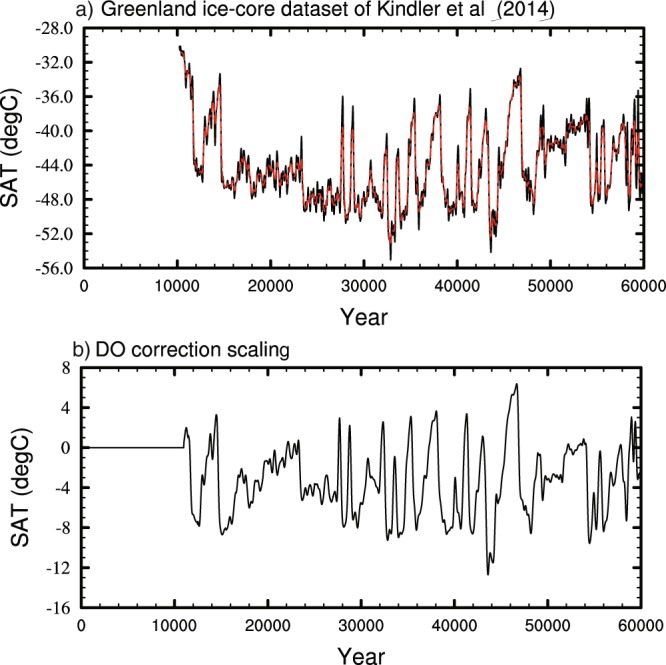
The Greenland ice-core dataset and the temporal correction used to incorporate millennial scale variability to the dataset. (**a**) The Kindler *et al*.^8^ dataset splined to a uniform 20-year timestep (black line) and following filtering using a 500-year low-pass filter (red line). (**b**) Difference in Greenland temperature between the splined data and the filtered ice-core dataset, this is used to scale a hosing experiment to give the spatial impact of millennial variability.

The first step in isolating these events was to take the Greenland ice-core temperature reconstruction from Kindler *et al*., (Fig. 6a)^8^. This temperature record covers the whole of the last glacial period (10–120 kyr) and is derived from δ^15^N isotopes incorporated with a firn densification (i.e. the compaction of the perennial snowpack) and heat diffusion model. This record provides the most reliable estimate of the abrupt temperature increase during DO events, and is not subject to seasonal biases that affect temperature reconstructions derived from water isotope records^8^. The Kindler record was splined using the same methodology as outlined above, to a uniform 20-year timestep from 10–60 kyr BP. This was then low-pass filtered (Fig. 6a; red line) to remove variability on timescales less than 500 years, which might conflict with the interannual variability applied in the previous step. The filtered timeseries is used to derive a temporal correction to the overall climate timeseries (Fig. 6b). The difference in Greenland temperature between the snapshot runs (splined to every year and then smoothed with 100 year running mean) and the Greenland ice-core temperatures is taken as a millennial-scale correction.

This correction was then used to scale a hosing experiment to give the spatial climate impact of the D-O events. The hosing experiment was performed using HadCM3B-M2.1 at 21 kyr, the period representing the last-glacial maximum. A freshwater forcing of 1 Sv is continually applied to the simulation (1 Sv = 1 × 10^6^ m^3^ s^−1^) over the Atlantic Ocean between 50°–70°N for 200 years. This acts to decrease the strength of the AMOC and causes a consequent climatic impact across the Northern Hemisphere centred around Greenland, with the final 50 years of the simulation used for analysis. Although this drives a cooling effect, it provides a spatial fingerprint for the millennial scale climate change. This approach is similar to that used in TraCE-21ka^48^, where the freshwater forcing was continually updated to match proxy records of Greenland temperature. We adjusted the hosing response to achieve the same model-data agreement.

**Fig. 7:**
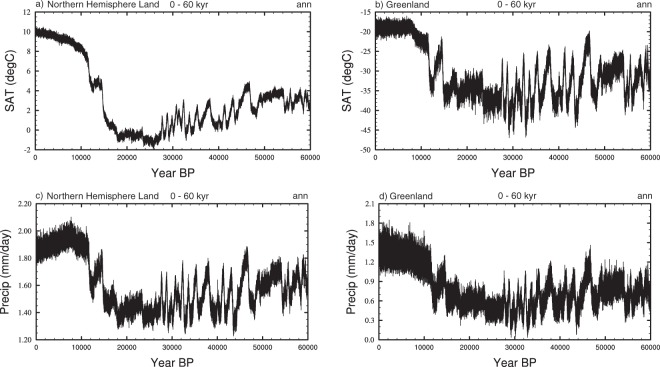
Timeseries showing the addition of DO variability to the splined and interannual timeseries. The onset and duration of D-O variability is identified from Kindler *et al*.^8^ and the spatial impact is identified via separate hosing experiments (see the text). (**a**) Northern Hemisphere SATs (°C). (**b**) Greenland SATs (°C), Northern Hemisphere precipitation (mm/day), and (**d**) Greenland precipitation (mm/day).

The resultant scaled extent and spatial patterns of the D-O events are then added to the splined and interannual data in 20-year segments (which is the approximate resolution of the Greenland ice-cores) from 11,000 to 60,000 years BP. This avoids any millennial-scale correction being applied during the Holocene. The consequent timeseries for the Northern Hemisphere and Greenland is shown in Fig. 7.

A limitation of this technique is that it assumes that all millennial scale variability is driven by changes in the Atlantic Meridional Overturning Circulation (AMOC). It is a similar approach to that used in the Trace-21Ka experiments. We also use only one LGM hosing experiment to derive the spatial fingerprint of millennial variability, despite there being a range of climate configurations present over the past 60 kyr. This decision reflects past unpublished work in which we carried out an ensemble of hosing experiments using HadCM3B that incorporated varying climate states representing the past 60 Kyr, in order to investigate the Heinrich events. These showed that that the spatial pattern of the hosing experiments does not vary depending on the climate state in the HadCM3B model. Finally, our overall approach does not include solar and volcanic forcing, as they are not included in the original snapshot GCM simulations.

#### Downscaling and bias correction

Following this, the dataset has been downscaled from the standard HadCM3B-M2.1 resolution (3.75° × 2.5°) to 0.5° resolution via bilinear interpolation. This has been performed using the NCL function linint2.

The final step is to bias correct the downscaled climate data. This has been done for temperature, precipitation, minimum monthly temperature and incoming shortwave energy. The bias corrected temperature data is also used to construct the wind-chill dataset. Snow-depth (as snow water equivalent) and number of rainy days per month have not been bias corrected.

The temperature (and consequently wind-chill), precipitation and minimum monthly temperature datasets have been bias corrected using the high-resolution CRU CL v2.0 observational dataset from the University of East Anglia, covering the period 1901 to 1990^32^ at 1/6^th^ degree resolution (1080 × 2160). This has been generated from over 10,000 temperature stations and 25000 precipitation stations. The dataset is first upscaled to 0.5° resolution. The incoming shortwave dataset has been bias corrected using the EWEMBI dataset^49^, which covers the period 1979–2013 on a 0.5° resolution. It is worth noting that these are not directly comparable observational datasets to use for bias correction, as our timeseries finishes with pre-industrial greenhouse gas concentrations. However in HadCM3B-M2.1 this has been shown to have only a small impact relative to the model biases^31^.

The differences between the observations and modelled values are calculated for each month and for each grid square. This correction is then applied to the whole time period, which assumes that the bias is constant throughout time. With precipitation, the ratio between the modelled and CRU dataset is calculated which is then multiplied with the modelled data. However, some areas that are very dry might show large differences in the ratio compared to observations, although actual precipitation amounts might be very small. In order to avoid very large scaling values, the bias correction scaling of precipitation is capped at three times the modelled value.

**Fig. 8:**
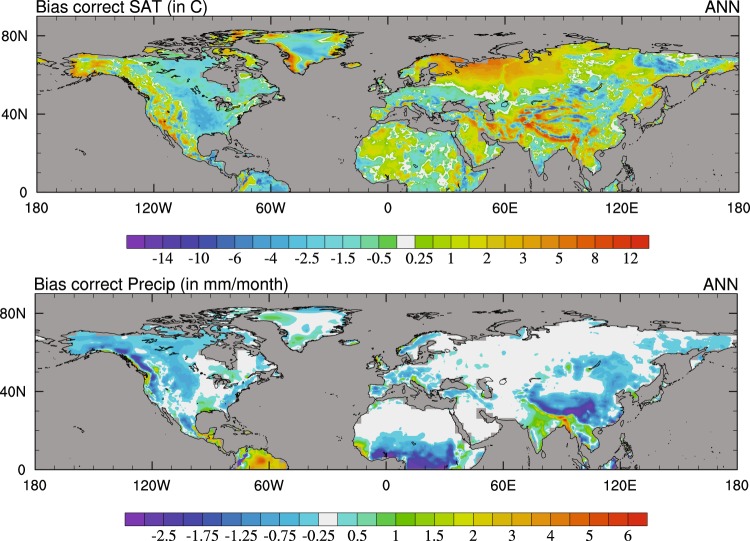
Mean annual bias corrections used to bias correct the model data. These have been calculated using the CRU data. (**a**) SATs (°C), and (**b**) precipitation (mm/month).

**Fig. 9:**
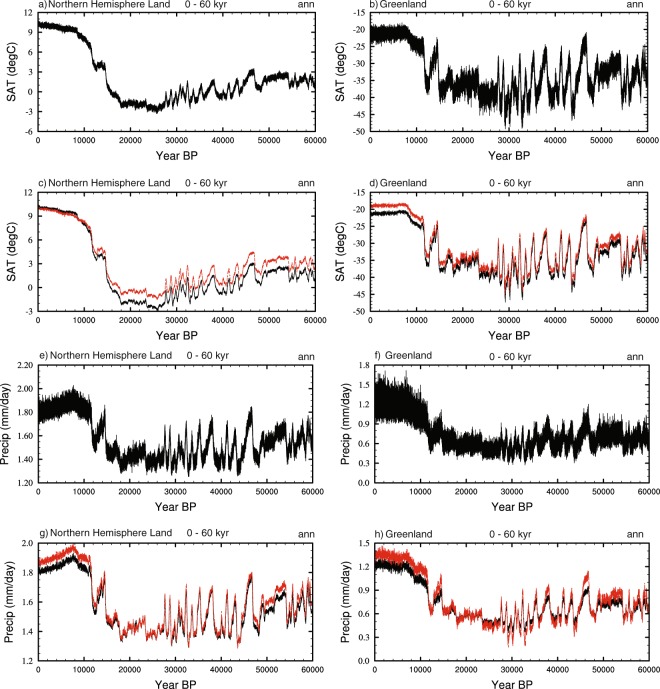
Timeseries showing the bias corrected data against the pre-bias corrected. (**a**) Bias corrected Northern Hemisphere SATs (°C), (**b**) bias corrected Greenland SATs (°C), (**c**) comparison of bias corrected (black) SATs (°C) against pre-bias corrected (red) SATs for the Northern Hemisphere smoothed with a 20-year running mean, (**d**) the same as (**c**) but for Greenland. (**e**–**h**) the same as (**a**–**d**) but for precipitation (mm/day).

The resultant mean annual corrections for temperature and precipitation are shown in Fig. 8. The final bias corrected timeseries for SAT and precipitation, compared with the pre-bias corrected data is shown in Fig. 9. In HadCM3B-M2.1, surface air temperatures are subject to a cold bias towards the poles, particularly over Russia and Scandinavia^31^. As such, there is a small increase in NH extra-tropical land temperature in the Holocene following bias correction. In Greenland, there is a small decrease in SATs due to a warm bias simulated in the model. The model does a reasonable job at simulating spatial patterns of precipitation, and is comparable to other CMIP5 models^31^. The model however overestimates precipitation in areas of significant topography, such as the Himalayas, Tibet and the Rockies, although negative biases in observations can act to amplify this^50^. These regions are likely to be the driver behind the decrease in NH precipitation following bias correction. The finalised datasets used to produce this plot can be found within the NERC digital repository^51^.

## Data Records

The datasets are in the form of NetCDF files and can be found within the NERC digital repository^51^. The climate variables and units are; temperature (°C), precipitation (mm/day), incoming shortwave energy (Wm^−2^), minimum monthly temperature (°C), snow depth as snow water (or liquid) equivalent (m), wind chill (°C) and number of rainy day per month (between 0–30). It is worth noting that the number of rainy days per month includes only those days when rain exceeds 0.4 mm/day. This is because daily rainfall in HadCM3B (like many GCMs) is prone to drizzle, i.e. rain every day, but does not show enough strong rainfall events.

Each climate variable is represented by a set of 24 files that have been compressed into seven folders: Temp, Precip, downSW, wchill, snowSWE, RainDays and minMonth. Each file represents 2500 years, equivalent to 30000 months, between the latitudes 0° to 90°N at 0.5° resolution. Each file therefore has the dimensions 180 (lat) × 720 (lon) × 30000 (month).

The seven folders contain the regridded, and with the exception of snow depth and rainy days, bias corrected data. The separate datasets for the different stages of the Methods (e.g. splining, addition of interannual variability and DO variability) are not given but are available on request. These are global datasets at the original HadCM3B-M2.1 resolution (2.75° × 3.25°).

We also provide the netcdf files for the land-sea mask and ice fraction, which have been compressed into two folders; landmask and IceFrac. These are provided as annual files, so have the dimensions 180 (lat) × 720 (lon) × 2500 (year).

We provide an example subset of the temperature data (“test_decadal_tas_0_2.5kyr.nc”), which gives decadal averages for each month for 0–2500 years. The scripts for each stage of the methodology (splining, interannual variability, millennial variability, bias correction and downscaling) are given in the Scripts folder.

There are a number of additional variables that have not yet been produced but could be generated on request; these are total evaporation (mm/day), soil carbon (KgCm^−2^) and soil moisture (Kgm^−2^). Note that data can only be produced for the Northern Hemisphere.

The naming convention for the bias corrected files (temperature, precipitation, wind-chill, minimum monthly temperatures and incoming shortwave) is:

bias_regrid_<variable>_<year start>_<year end>

And for the remaining climate variables (snow depth and rainy days), the land-sea mask and ice-fraction is:

regrid_<variable>_<year start>_<year end>

variable: tas = surface air temperature

pr = precipitation

surface_downwelling_shortwave_flux = Incoming shortwave energy

wchill = wind-chill

tempmonmin_abs = minimum monthly temperature

lwe_thickness_of_surface_snow_amount = Snow (liquid) water equivalent

number_rainy_days = number of rainy days per month (>0.4 mm/day threshold)

landmask = land-sea mask

icefrac = ice fraction

year start and year end: these refer to the beginning and end years of the file, and comprise a 2500 year section between 0 and 60000 years.

## Technical Validation

A broad validation of the HadCM3B-M2.1 model against observational datasets is given in section 5 of Valdes *et al*.^31^. They show that the model reproduces an accurate representation of different aspects of the climate system in land and sea surface temperatures, precipitation and ocean circulation. Similarly, they show that HadCM3B-M2.1 outperforms many of the CMIP5 models, particularly when evaluating surface air temperatures (see their Fig. 2), despite the cold bias in the model as discussed above. Here we provide a comprehensive validation for the temperature and precipitation datasets.

### Timeseries validation

**Fig. 10:**
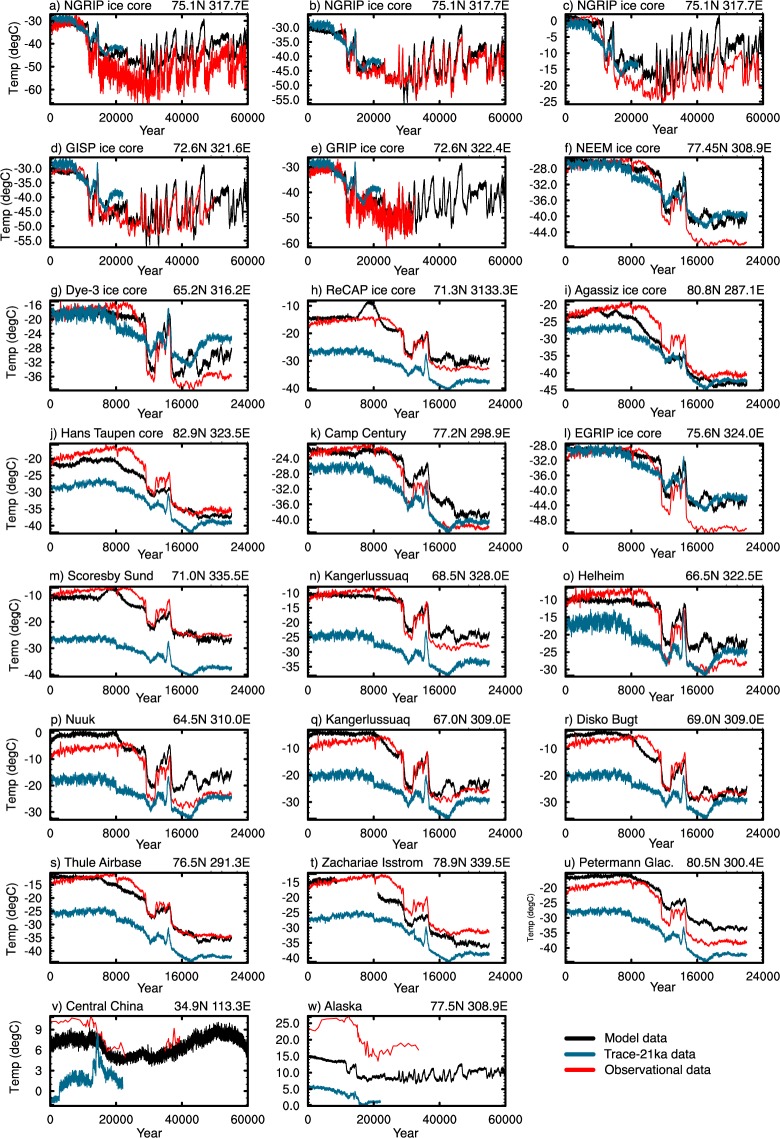
Validation timeseries plots for SATs (°C). The bias corrected temperatures (black) are shown with a range of observational datasets (red). These are shown with the Trace-21Ka data in blue^48^; note this this has not been bias corrected. References for panels (a–e,v,w) are given in the study of Shakun *et al*. (2012)^8,52–56^, references for the remaining 16 datasets are given in the supplementary data of Buizert *et al*.^57^. The associated lat/lon position for each dataset is given above each panel. Note the longer time range in panels (a–e,v and w).

Validating palaeo-climate timeseries against observations is challenging due to the lack of observational datasets, which are themselves subject to a range of biases and uncertainties. Here we compare temperature and precipitation against a range of available ice-core and land-based datasets to validate in the temporal domain. Figure 10 shows 23 land-based temperature records spanning the last glacial period from ice-cores (a–l), the ice margin (o–u) and at locations away from the ice-sheet (v–w), against the modelled temperature smoothed with a 20-year running mean. The references for panels a–e, v and w are given in the study of Shakun *et al*. (2012)^8,52–56^, whist references for the remaining 16 datasets are given in the supplementary data of Buizert *et al*.^57^. Each timeseries are shown with the TraCE-21ka^48^ dataset as the blue line, note here however that this latter model dataset has not been bias-corrected.

Panels a–e compare against the three Greenland ice-cores; GRIP, NGRIP, and GISP. There is generally good correlation between all three of these cores. The NGRIP datasets of Rasmussen *et al*., (2006; panel a)^55^ and Masson-Delmotte *et al*., (2005; panel c)^9^ show colder temperatures throughout the period of between 6 °C to 10 °C. Note here that the Masson-Delmotte *et al*.^9^ dataset is shown as anomalies from present-day. This discrepancy is reduced following re-calibration of the dataset in the study of Kindler *et al*. (panel b), which is very well correlated to our dataset due to the fact this was used to normalise our D-O events. There is also good correlation between the patterns of temperature change and absolute values with both the GISP and GRIP cores, although there is a greater decline in Holocene temperatures in the modelled data.

The remaining ice-core datasets, located across Greenland and Arctic Canada, show generally good agreement with the modelled data, particularly for the Bølling Allerød and Younger Dryas periods. However, compared to the Agassiz and Hans Tausen ice cores, the modelled temperatures are too cool in the Holocene, whilst in the Cam Century and EGRIP ice cores, modelled LGM temperatures are warmer than the ice core datasets. Similarly, the ice-margin datasets generally show good comparison with the modelled data. There is also evidence from a number of these timeseries that the model simulates the Holocene Climatic Optimum (HCO), albeit to a lesser degree that many of the observational datasets (specifically panels j, k, p, q and r). This discrepancy in the modelled and proxy data during this period has been shown to be the case in a number of other climate models^58^. This may reflect biases across the current generation of models and/or biases with the reconstructions themselves.

Panels (v) and (w) are the only available datasets that permit validation away from the Greenland ice-sheet. Panel (v) shows mean July air temperatures derived from fossil assemblages of Chironomidae from burial lakes in Alaska^53^. There are greater discrepancies here than the ice-core/margin temperatures, however the general pattern of temperature change over the period is consistent, specifically the spike in temperatures at the Holocene followed by a decline until the LGM. Pre-industrial temperatures however are lower in the modelled output by up to 5 °C.

Panel (w) shows surface air temperatures reconstructed from membrane lipids of soil bacterial from the Mangshan loess plateau in Central China. Here there is significant discrepancy between the two datasets, with modelled data being up to 16 °C different. Although no past studies have investigated this region using HadCM3B-M2.1, other models investigating current day conditions have highlighted the difficulty in modelling climate across this region^59^. This is in part due to its complex topography and location between the semi-arid continental region to the west, and the humid monsoon regions to the east. This may be a region of uncertainty with the dataset, which may be exacerbated by the bi-linear interpolation technique used to downscale the data. Using a more advanced statistical downscaling technique, as shown in past studies, may improve prediction skill^60^.

**Fig. 11:**
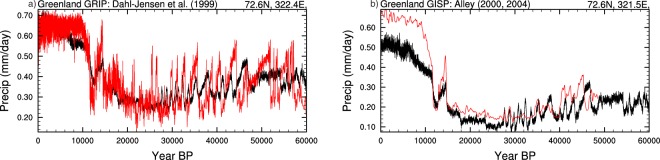
Validation plots for precipitation (mm/day). The bias corrected precipitation (black) is shown against two observational datasets from Greenland (red). Both of these datasets represent snow accumulation, which is used as an equivalent to precipitation in the absence of total precipitation datasets. (**a**) Shows the GRIP ice-core and (**b**) the GISP Ice-core. The associated reference and lat/lon position for each dataset is given above each panel. Mismatches in the timing of abrupt events are due to updates to the Greenland ice-core chronologies since the publication of these two records.

Validating palaeo-precipitation is more challenging due to the lack of observational datasets. Here we compare the precipitation timeseries against reconstructions of snow accumulation, derived from the GRIP and GISP ice-cores^61–63^ (Fig. 11). Although this is not a direct comparison, the extremely low temperatures mean that the influence of rainfall during the glacial period was minimal. There is generally good correlation between the model and GRIP observational datasets, particularly between 0–30 kyr BP. The GISP dataset however shows greater than modelled precipitation in the Holocene, but there is good agreement between the Younger-Dryas to approximately 40 kyr BP. Both datasets show a breakdown in correlation towards the end of the datasets, where there is some disagreement of the timings of the DO events, which is likely related to differences in ice-core chronologies. The agreement between the model and the ice-core records in terms of the magnitude of accumulation increase across multiple DO events lends support to the overall approach we have employed.

### Spectral validation

**Fig. 12:**
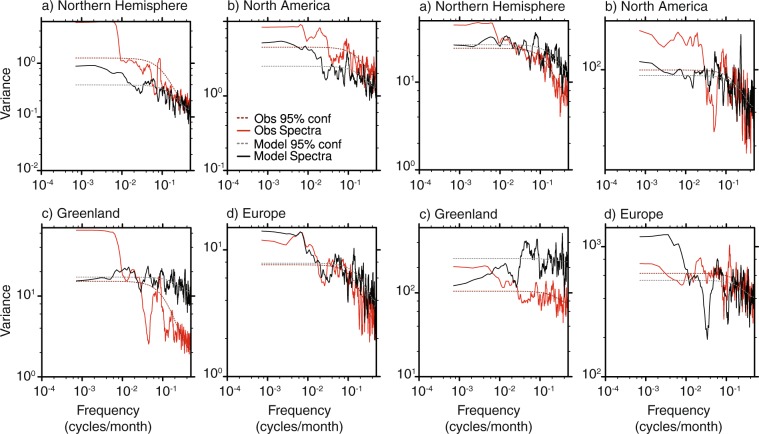
Power spectrum of the CRU observational dataset spanning 1900–2017 and the final 117 years of the model dataset for four different regions. The series mean, least squares linear trend, annual and seasonal cycles have been removed prior to analysis. (**a**–**d**) show temperature with CRU in red and the model in black, (**e**–**h**) show precipitation. The dashed lines represent the 95% confidence level determined by a red noise spectrum of a first-order auto-regressive (AR1) process.

Figure 12 shows the power spectra for the final 117 years of the model data again the CRU observational dataset (1900–2017)^64^ for temperature (left panels) and precipitation (right panels), in order to compare the spectral characteristics. The series mean and least squares linear trend have been removed prior to analysis, in addition to the annual and seasonal cycles in order to remove peaks equivalent to the 12-month and 6-month periods. Note that the seasonal cycle is well represented in the model data for both temperature and precipitation. The data is shown in a log-log convention in order to highlight low frequency variability. Although these datasets are not directly comparable as they represent different time periods, past work^31^ has shown that the biases associated with this are small compared to the model biases.

The spectral plots show a significant degree of noise in both the observational and modelled datasets over the period of analysis, which may reflect the short timescale analysed. There are a number of differences in the regional power spectra for temperature, particularly in Greenland. However, North American and European temperature spectra show some similarities, particularly an approximate 10-month peak in North America and a lower frequency peak in Europe of between 11 and 12 years. This low frequency peak may represent a mode of North Atlantic variability such as the Atlantic Multidecadal Oscillation.

Variability in regional precipitation also shows a significant degree of noise for both observations and modelled data. The spectra for whole Northern Hemisphere region shows greater parity however, with potential peaks present for both datasets at approximately 13 to 14 years and at 5 to 6 years.

### Spatial validation

**Fig. 13:**
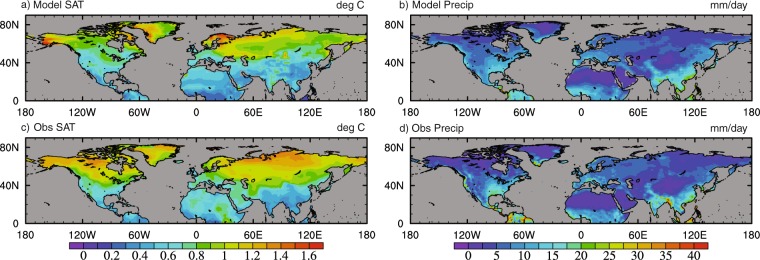
Spatial pattern of the standard deviation for the final 117 years of the model dataset and the CRU observational dataset spanning 1900–2017. (**a**) Model SATs (°C), (**b**) CRU SATs (°C), (**c**) model precipitation (mm/day) and (**d**) CRU precipitation (mm/day).

**Fig. 14:**
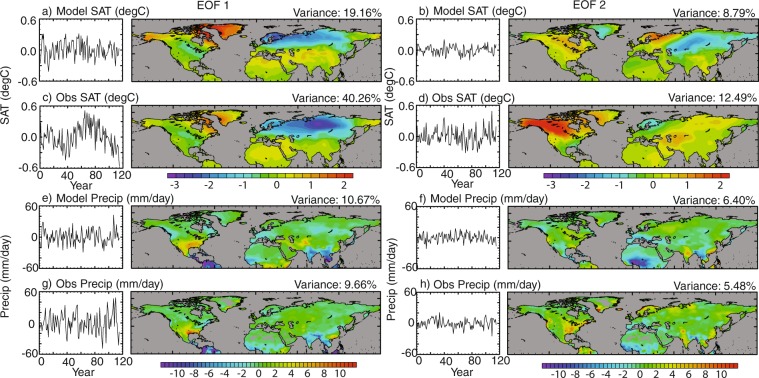
First and second EOFs showing the spatial scale of variability and the corresponding principal component timeseries of the CRU observational dataset spanning 1900–2017 and the final 117 years of the model dataset. The left panels show EOF1 and the right panels EOF2, for temperature (**a**–**d**) and precipitation (**e**–**h**).

In order to assess if the spatial scale of the modelled data is comparable with observations, Figs 13 and 14 show the annual standard deviation, and the first and second EOFs of northern hemisphere winter of the modelled data against the CRU dataset^64^. The CRU data spans the period 1900–2017, which is compared with the final 117 years of the data.

Generally there is good agreement in the annual mean standard deviations (SD) for both temperature and precipitation, and the major patterns of climate are well represented. In some regions however, variability is modelled to be too large, such as Scandinavia and Alaska, which may reflect the cold bias in the regions and so an exaggerated seasonal cycle. In contrast, other regions show that the modelled variability is too small, such as across Northern Russia, Canada, Northern Africa and the Middle East. Precipitation also shows good similarities with observed data, although variability is too small in regions of the tropics, and too large in some very dry regions such as the Sahara/Sahel, the Mongolian Plateau and Greenland.

The first EOF (Fig. 14) for both modelled and observational SAT show good similarities, and may represent the pattern of the Arctic Oscillation (AO), with regions of variability over Greenland and an opposing pattern over Scandinavia and Northern Asia. The centres of variability differ between the datasets, specifically the extent of the eastward region of variance in observations. This oscillation accounts for a greater proportion of the variance in the observational dataset, and may also be evident in the principal component (PC) timeseries that shows variability on multi-decadal timescales. The second EOFs show greater disparity and are harder to interpret, with an area of variability over North America in the modelled data that may represent a positive phase of the Pacific/North American (PNA) teleconnections pattern which is not represented in the modelled data. Again there is a possible decadal scale oscillation apparent in the PC timeseries for the observational dataset that is not as well represented in the modelled data.

With precipitation, there is generally good correlation between the annual EOF1 and EOF2 against observations, highlighting strong opposing areas of variance in south-west USA and tropical South America, and across India and South-East Asia. This may be linked to the zonal shift in the position of the ITCZ at annual timescales. EOF2 also shows similarities, and variability may be linked to monsoon precipitation, including the African Monsoon (AFM) and Asian and Indo-Pacific monsoon (AIPM). The EOFs presented here are similar to those generated from the re-forecast data of Zuo *et al*.^65^.

Both Figs 13 and 14 indicate that spatially, on high frequency annual/seasonal timescales, the spread and variability of the model data is broadly consistent with observations.

## Usage Notes

The timeseries NetCDF files are in 2500 year/30000 month sections from 0 BP, i.e. the year 1950. Therefore the first month in the 0–2.5 kyr data files represents January 1950. Pre-industrial greenhouse gas concentrations are used to represent this year, as shown in Table 1. Temperature is given in units of °C, precipitation is in mm/month, incoming shortwave energy in Wm^−2^, minimum monthly temperature in °C, snow depth as snow water (or liquid) equivalent in m, wind chill in °C, and number of rainy days per month (with a threshold of 0.4 mm/day) as a value between 0–30. Note also that for the snow-depth dataset, a mask is applied to all grid squares covered in an ice-sheet, so snow depth is only shown for ice-free grid-squares.

HadCM3B-M2.1 and the methodology used here is subject to a range of uncertainties and biases that inherently influences the simulated climatologies. A number of these are outlined in the above text, but there is a wide range of other literature that gives an overview of these uncertainties both in the model^31^, and the limitations associated with the methodology^2^.

HadCM3B-M2.1 is constructed on a Cartesian grid, so when calculating global or regional means a weighting must be applied to the data that takes into account the smaller size of grid squares towards the poles. The data can be used with a wide range of post processing software.

## Data Availability

Raw model output is available for further analysis from https://www.paleo.bristol.ac.uk/ummodel/scripts/papers/Armstrong_et_al_2019.html. A list of the snapshot simulation names used in this experiment is given in Table 1. All scripts used to construct the climate timeseries have been written using the NCAR command language (NCL, Version 6.4.0) and are available within the NERC digital repository^51^.

## References

[1] Masson-Delmotte V (2006). Past temperature reconstructions from deep ice cores: relevance for future climate change. Clim Past.

[2] Singarayer JS, Valdes PJ (2010). High-latitude climate sensitivity to ice-sheet forcing over the last 120 kyr. Quaternary Sci Rev.

[3] Clark PU, Mix AC (2002). Ice sheets and sea level of the Last Glacial Maximum. Quaternary Sci Rev.

[4] Rahmstorf S (2002). Ocean circulation and climate during the past 120,000 years. Nature.

[5] Pelto BM, Caissie BE, Petsch ST, Brigham-Grette J (2018). Oceanographic and Climatic Change in the Bering Sea, Last Glacial Maximum to Holocene. Paleoceanogr Paleocl.

[6] Goebel T, Waters MR, O’Rourke DH (2008). The Late Pleistocene dispersal of modern humans in the Americas. Science.

[7] Jouzel J (2007). Orbital and millennial Antarctic climate variability over the past 800,000 years. Science.

[8] Kindler P (2014). Temperature reconstruction from 10 to 120 kyr b2k from the NGRIP ice core. Clim Past.

[9] Masson-Delmotte V (2005). GRIP deuterium excess reveals rapid and orbital-scale changes in Greenland moisture origin. Science.

[10] Wolff EW, Chappellaz J, Blunier T, Rasmussen SO, Svensson A (2010). Millennial-scale variability during the last glacial: The ice core record. Quaternary Sci Rev.

[11] Roche DM, Dokken TM, Goosse H, Renssen H, Weber SL (2007). Climate of the Last Glacial Maximum: sensitivity studies and model-data comparison with the LOVECLIM coupled model. Clim Past.

[12] Yan M, Wang B, Liu J (2016). Global monsoon change during the Last Glacial Maximum: a multi-model study. Clim Dynam.

[13] Yanase W, Abe-Ouchi A (2007). The LGM surface climate and atmospheric circulation over East Asia and the North Pacific in the PMIP2 coupled model simulations. Clim Past.

[14] Berger A, Loutre MF, Gallee H (1998). Sensitivity of the LLN climate model to the astronomical and CO2 forcings over the last 200 ky. Clim Dynam.

[15] Tarasov L, Peltier WR (1999). Impact of thermomechanical ice sheet coupling on a model of the 100 kyr ice age cycle. J Geophys Res-Atmos.

[16] Kutzbach JE, Liu XD, Liu ZY, Chen GS (2008). Simulation of the evolutionary response of global summer monsoons to orbital forcing over the past 280,000 years. Clim Dynam.

[17] Jackson CS, Broccoli AJ (2003). Orbital forcing of Arctic climate: mechanisms of climate response and implications for continental glaciation. Clim Dynam.

[18] Lorenz SJ, Lohmann G (2004). Acceleration technique for Milankovitch type forcing in a coupled atmosphere-ocean circulation model: method and application for the holocene. Clim Dynam.

[19] Hoogakker BAA (2016). Terrestrial biosphere changes over the last 120 kyr. Clim Past.

[20] Swindles, G. T., Morris, P. J., Baird, A. J., Blaauw, M. & Plunkett, G. Ecohydrological feedbacks confound peat-based climate reconstructions. *Geophys Res Lett* **39**, 10.1029/2012gl051500 (2012).

[21] Giampoudakis K (2017). Niche dynamics of Palaeolithic modern humans during the settlement of the Palaearctic. Global Ecol Biogeogr.

[22] Nogues-Bravo D (2016). Amplified plant turnover in response to climate change forecast by Late Quaternary records. Nat Clim Change.

[23] Stringer, C. *et al*. In *Neanderthal and modern humans in the European landscape of the last glaciation: archaeological results of the Stage 3 Project* (eds van Andel, T. H. & Davies, W.) 233–240 (Oxbow books, 2004).

[24] Clark PU, Pisias NG, Stocker TF, Weaver AJ (2002). The role of the thermohaline circulation in abrupt climate change. Nature.

[25] Dansgaard W (1993). Evidence for General Instability of Past Climate from a 250-Kyr Ice-Core Record. Nature.

[26] Elliot M, Labeyrie L, Duplessy JC (2002). Changes in North Atlantic deep-water formation associated with the Dansgaard-Oeschger temperature oscillations (60–10 ka). Quaternary Sci Rev.

[27] Heinrich H (1988). Origin and Consequences of Cyclic Ice Rafting in the Northeast Atlantic-Ocean during the Past 130,000 Years. Quaternary Res.

[28] Birchfield GE, Broecker WS (1990). A Salt Oscillator in the Glacial Atlantic? 2. A “Scale Analysis” Model. Paleoceanography.

[29] Broecker WS (2000). Abrupt climate change: causal constraints provided by the paleoclimate record. Earth-Sci Rev.

[30] Rehfeld K, Munch T, Ho SL, Laepple T (2018). Global patterns of declining temperature variability from the Last Glacial Maximum to the Holocene. Nature.

[31] Valdes PJ (2017). The BRIDGE HadCM3 family of climate models: HadCM3@Bristol v1.0. Geosci Model Dev.

[32] New M, Lister D, Hulme M, Makin I (2002). A high-resolution data set of surface climate over global land areas. Climate Res.

[33] Pope VD, Gallani ML, Rowntree PR, Stratton RA (2000). The impact of new physical parametrizations in the Hadley Centre climate model: HadAM3. Clim Dynam.

[34] Gordon C (2000). The simulation of SST, sea ice extents and ocean heat transports in a version of the Hadley Centre coupled model without flux adjustments. Clim Dynam.

[35] Cox PM (1999). The impact of new land surface physics on the GCM simulation of climate and climate sensitivity. Clim Dynam.

[36] Essery, R. L. H., Best, M. J., Betts, R. A., Cox, P. M. & Taylor, C. M. Explicit representation of subgrid heterogeneity in a GCM land surface scheme. *J Hydrometeorol* **4**, 530–543, doi:10.1175/1525-7541(2003)004%3C0530:Eroshi%3E2.0.Co;2 (2003).

[37] Semtner, A. J. Model for Thermodynamic Growth of Sea Ice in Numerical Investigations of Climate. *J Phys Oceanogr* **6**, 379–389, doi:10.1175/1520-0485(1976)006%3C0379:Amfttg%3E2.0.Co;2 (1976).

[38] Loulergue L (2008). Orbital and millennial-scale features of atmospheric CH4 over the past 800,000 years. Nature.

[39] Petit JR (1999). Climate and atmospheric history of the past 420,000 years from the Vostok ice core, Antarctica. Nature.

[40] Spahni R (2005). Atmospheric methane and nitrous oxide of the late Pleistocene from Antarctic ice cores. Science.

[41] Peltier WR (2004). Global glacial isostasy and the surface of the ice-age earth: The ice-5G (VM2) model and grace. Annu Rev Earth Pl Sc.

[42] Martinson DG (1987). Age Dating and the Orbital Theory of the Ice Ages - Development of a High-Resolution-0 to 300,000-Year Chronostratigraphy. Quaternary Res.

[43] Davies-Barnard T, Ridgwell A, Singarayer J, Valdes P (2017). Quantifying the influence of the terrestrial biosphere on glacial-interglacial climate dynamics. Clim Past.

[44] Wessel P, Bercovici D (1998). Interpolation with splines in tension: A Green’s function approach. Math Geol.

[45] Ditlevsen PD, Svensmark H, Johnsen S (1996). Contrasting atmospheric and climate dynamics of the last-glacial and Holocene periods. Nature.

[46] Peltier WR, Vettoretti G (2014). Dansgaard-Oeschger oscillations predicted in a comprehensive model of glacial climate: A “kicked” salt oscillator in the Atlantic. Geophys Res Lett.

[47] Hopcroft PO, Valdes PJ, Beerling DJ (2011). Simulating idealized Dansgaard-Oeschger events and their potential impacts on the global methane cycle. Quaternary Sci Rev.

[48] Liu Z (2009). Transient Simulation of Last Deglaciation with a New Mechanism for Bolling-Allerod Warming. Science.

[49] Lange S (2019). EartH2Observe, WFDEI and ERA-Interim data Merged and Bias-corrected for ISIMIP (EWEMBI).

[50] Adam JC, Clark EA, Lettenmaier DP, Wood EF (2006). Correction of global precipitation products for orographic effects. J Climate.

[51] Armstrong E, Hopcroft PO, Valdes P (2019). Centre for Environmental Data Analysis.

[52] Cuffey KM, Clow GD (1997). Temperature, accumulation, and ice sheet elevation in central Greenland through the last deglacial transition. J Geophys Res-Oceans.

[53] Kurek J, Cwynar LC, Ager TA, Abbott MB, Edwards ME (2009). Late Quaternary paleoclimate of western Alaska inferred from fossil chironomids and its relation to vegetation histories. Quaternary Sci Rev.

[54] Peterse F (2011). Decoupled warming and monsoon precipitation in East Asia over the last deglaciation. Earth Planet Sc Lett.

[55] Rasmussen, S. O. *et al*. A new Greenland ice core chronology for the last glacial termination. *J Geophys Res-Atmos* **111**, 10.1029/2005jd006079 (2006).

[56] Shakun JD (2012). Global warming preceded by increasing carbon dioxide concentrations during the last deglaciation. Nature.

[57] Buizert C (2018). Greenland-Wide Seasonal Temperatures During the Last Deglaciation. Geophys Res Lett.

[58] Liu ZY (2014). The Holocene temperature conundrum. P Natl Acad Sci USA.

[59] Wang L, Cheung KKW, Tam CY, Tai APK, Li YB (2018). Evaluation of the Regional Climate Model over the Loess Plateau of China. Int J Climatol.

[60] Peng SZ, Gang CC, Cao Y, Chen YM (2018). Assessment of climate change trends over the Loess Plateau in China from 1901 to 2100. Int J Climatol.

[61] Alley RB (2000). The Younger Dryas cold interval as viewed from central Greenland. Quaternary Sci Rev.

[62] Alley, R. B. In *Data Contribution Series #2004*–*013* (ed. IGBP PAGES/World Data Center for Paleoclimatology) (NOAA/NGDC Paleoclimatology Program, Boulder CO, USA, 2004).

[63] Dahl-Jensen, D., Johnsen, S., Hammer, C. U., Clausen, H. B. & Jouzel, J. In *Ice in the Climate System* (ed. Peltier, W. R.) 517–532 (Springer-Verlag, 1993).

[64] Harris I, Jones PD, Osborn TJ, Lister DH (2014). Updated high-resolution grids of monthly climatic observations - the CRU TS3.10 Dataset. Int J Climatol.

[65] Zuo ZY (2013). Predictable patterns and predictive skills of monsoon precipitation in Northern Hemisphere summer in NCEP CFSv2 reforecasts. Clim Dynam.

