# Author Correction: A simulated Northern Hemisphere terrestrial climate dataset for the past 60,000 years

**DOI:** 10.1038/s41597-020-0432-8

**Published:** 2020-03-10

**Authors:** Edward Armstrong, Peter O. Hopcroft, Paul J. Valdes

**Affiliations:** 1 School of Geographical Sciences, University of Bristol, Bristol, UK; 2 Cabot Institute, University of Bristol, Bristol, UK; 3 School of Geography, Earth and Environmental Sciences, University of Birmingham, Edgbaston, B15 2TT UK

Correction to: *Scientific Data* 10.1038/s41597-019-0277-1, published online 07 November 2019

Following publication of this Data Descriptor, a number of issues in the associated dataset hosted at Centre for Environmental Data Analysis have been subsequently identified and rectified. The changes made are as follows:

- The time dimension is now going forward in time. For example, for the 0-2.5kyr data file, the first time slice is Jan 2500 BP and the last is December 0 BP. The text of the Usage Notes section has been updated in both the HTML and PDF versions to reflect this change.
- The data have been re-gridded onto a new longitude coordinate system. The longitude grid now spans -180 to 179.5
- The files have now been compressed and are now in short format.
- A number of instances where the months were incorrectly organised have been corrected.

None of the values or figures presented in the paper are affected by these changes.

The revised dataset has been assigned a new DOI (10.5285/4ca242208e904efe830af45f1697f730) and the corresponding citation has been updated in both the HTML and PDF versions.

